# Comprehensive analysis of autophagy associated genes and immune infiltrates in cervical cancer

**DOI:** 10.22038/IJBMS.2024.74431.16168

**Published:** 2024

**Authors:** Shuzhen Li, Kun Gao, Desheng Yao

**Affiliations:** 1 Department of Gynecologic Oncology, Guangxi Medical University Cancer Hospital Nanning, Guangxi Zhuang Autonomous Region 530021, PR China

**Keywords:** Autophagy, Bioinformatics, Immune Infiltrates, Mitogen-activated protein - kinase 3, Uterine cervical neoplasms

## Abstract

**Objective(s):**

Cervical cancer (CC) is the most common gynecological malignant tumor and the fourth leading cause of cancer-related death in women. The progression of CC is significantly affected by autophagy. Our objective was to use bioinformatics analysis to explore the expression, prognostic significance, and immune infiltration of autophagy-related genes in CC.

**Materials and Methods:**

We identified a set of autophagy-related differentially expressed genes (ARDEGs) from The Cancer Genome Atlas (TCGA) and Gene Expression Omnibus (GEO) databases. ARDEGs were further validated by The Human Protein Atlas (HPA), GSE52903, and GSE39001 dataset. Hub genes were found by the STRING network and Cytoscape. We performed Gene Set Enrichment Analysis (GSEA), Gene ontology analysis (GO), Kyoto Encyclopedia of Genes and Genomes (KEGG) analysis, and immune infiltration analysis to further understand the functions of the hub genes. Kaplan-Meier (K-M) and receiver operating characteristic (ROC) were used to check the hub genes.

**Results:**

A total of 10 up-regulated (CXCR4, BAX, SPHK1, EIF2AK2, TBK1, TNFSF10, ITGB4, CDKN2A, IL24, and BIRC5) and 19 down-regulated (PINK1, ATG16L2, ATG4D, IKBKE, MLST8, MAPK3, ERBB2, ULK3, TP53INP2, MTMR14, BNIP3, FOS, CCL2, FAS, CAPNS1, HSPB8, PTK6, FKBP1B , and DNAJB1) ARDEGs were identified. The ARDEGs were enriched in cell growth, apoptosis, human papillomavirus infection, and cytokine-mediated. Then, we found that low expression of MAPK3 was associated with poor prognosis in CC patients and was significantly enriched in immune pathways. In addition, the expression of MAPK3 was significantly positively correlated with the infiltration levels of macrophages, B cells, mast cell activation, and cancer-associated fibroblasts. Furthermore, MAPK3 was positively correlated with LGALS9, and negatively correlated with CTLA4 and CD40.

**Conclusion:**

Our results show that MAPK3 can be used as a new prognostic biomarker to predict the prognosis of patients with CC.

## Introduction

Cervical cancer (CC) is the most gynecological malignant tumor and the fourth leading factor in female cancer-related death (1). Although new therapeutic approaches, such as immunotherapy and vascular targeting therapy, have increased survival, the prognosis for patients with metastatic or recurring cancer is still dismal. The five-year overall survival rate for patients with advanced-stage CC is no higher than 40%(2). Therefore, it is crucial to explore new biomarkers for CC diagnosis, prognosis, and treatment.

Autophagy is a natural process in which the body breaks down and removes macromolecules and damaged structures while recycling cell materials (3). It is a significant biological mechanism involved in homeostasis, development, cell function, cell stress, and cytoplasmic recycling (4). Furthermore, this biological process is intricately linked to the development of tumors, as cancer cells rely on the essential nutrients and essential raw materials supplied by the host’s systemic autophagy to sustain their uncontrolled growth (5). Studies have shown that abnormal autophagy is related to various malignant tumors, such as lung carcinoma (6), cervical cancer (7), renal cell cancer (8), and liver cancer (9). In addition, autophagy is thought to be involved in tumor recurrence and treatment-resistance events (10). For example, MAP4K4 induces autophagy to reduce the chemosensitivity of CC (11), CDK6 can regulate glycolysis and autophagy in cervical cancer cells through mTORC1 independent of pRB (12), STAT3 plays a role in promoting cancer and anti-autophagy in CC (13). According to these studies, autophagy-related genes may be a reliable diagnostic and therapeutic biomarker for CC patients. Nonetheless, autophagy plays a variety of roles in tumor therapy, contingent upon the kind of cell, the surrounding milieu, and the stage of tumor progression.

Therefore, this study aims to identify autophagy-related genes (ARGs) and their biological functions in CC through comprehensive bioinformatics analysis. A set of ARDEGs were identified from TCGA and GEO databases. Hub genes were found using the STRING network and Cytoscape. Kaplan-Meier and ROC were used to check the hub genes. To further understand the function of hub genes, GO, KEGG, GSEA, and immune infiltration analysis were performed. Our results indicate that MAPK3 and BAX play a crucial role in the development of CC. This study provided new biomarkers for CC diagnosis, prognosis, and treatment.

## Materials and Methods

***Date download and procession***

TCGA-CESC expression profile data were extracted from TCGA using the TCGA biolink R package (14). We gathered 309 CC samples (cancer group, CESC) and 3 neighboring samples (Normal group, Normal) after excluding samples lacking crucial clinical information. The samples were normalized to Fragments Per Kilobase Million (FPKM). Normalization of count sequencing data in the TCGA-CESC dataset was performed utilizing the line package (15).

We downloaded the CESC related data sets GSE63514 (16), GSE7803 (17), GSE52903 (18), and GSE39001 (19) from the GEO database (20) via the R package “GEOquery” (21) and normalized the data again using the normalizeBetweenArrays function of the limma package. The gene expression values of the GSE63514 dataset from 28 CC patient samples and 24 matched adjacent normal tissue samples were identified using the GPL570 [HG-U133_Plus_2] Affymetrix Human Genome U133 Plus 2.0 Array platform. The gene expression values of the GSE7803 dataset were detected using the GPL96 [HG-U133A] Affymetrix Human Genome U133A Array platform, along with microarray gene expression profiling data from 21 CC patient samples and 10 partially matched adjacent normal tissue samples. The gene expression values of the GSE52903 dataset were detected using the GPL6244 [HuGene-1_0-st] Affymetrix Human Gene 1.0 ST Array [transcript (gene) version], along with microarray gene expression profiling data from 55 CC patient samples and 17 normal tissue samples. The gene expression values of the GSE39001 dataset were detected using the GPL6244 [HuGene-1_0-st] Affymetrix Human Gene 1.0 ST Array [transcript (gene) version], along with microarray gene expression profiling data from 19 CC patient samples and 5 normal tissue samples. All samples utilized in the analysis were derived from Homo sapiens. The annotation of dataset probe names was carried out by utilizing the corresponding GPL platform file. Additionally, all three GEO datasets were employed as validation sets. The dataset information is listed in Table 1. Finally, in order to obtain autophagy-related differential genes, we downloaded 222 ARGs from the HAMdb ( http://hamdb.scbdd.com/) (22) (Supplementary Table S1).

***Date procession***

Batch effects were removed from the CESC datasets GSE63514 and GSE7803 using the R package sva. To assess the impact of the batch effect, distribution, box plots, and principal component analysis (PCA) were employed to compare datasets before and after its adjustment.

***Cervical cancer-related differentially expressed genes***

We used the line package to investigate DEGs in normal and CESC tissues from the GSE63514 and GSE7803 datasets. DEGs were identified by screening with |log2(FC)|> 1 and *P*<0.05. LogFC>1 and *P*<0.05 represented up-regulated genes, while logFC<-1 and *P*<0.05 represented down-regulated genes among these DEGs. To obtain ARDEGs associated with CESC, we intersected the DEGs of the GSE63514 and GSE7803 datasets with the ARGs and generated a Venn diagram. Differential expressions are presented as volcano plots generated using the R package ggplot2 and heat map generated using the R package ComplexHeatmap [2.13.1](23).

***GO and KEGG***

GO (24) is widely used to perform comprehensive functional enrichment analyses on biological processes (BP), molecular functions (MF), and cellular components, making it a popular method in this field. KEGG (25) encompasses information about genomes, biological pathways, diseases, and drugs, providing a valuable resource in these areas. Utilizing the R package cluster Profiler (26), GO and KEGG annotation analysis were conducted on the up-regulated and down-regulated ARDEGs (26). *P*<0.05 was set as the threshold value for substantial enrichment, while *P*<0.05 and an FDR value (q.value) <0.05 were set as the cutoff criteria for significant enrichment. The *P*-values were corrected using the Benjamini-Hochberg (BH) method.

***Creating PPI networks and identifying hub genes***

A Protein-Protein Interaction (PPI) network of the ARDEGs was constructed using the STRING database (www.string-db.org). The criterion was regarded as the required minimum score of 0.15. With MCODE integrated into Cytoscape, hub genes were further discovered.

***Survival analysis of hub genes in cervical cancer***

Prognosis Kaplan-Meier (KM) curve analysis, a technique widely known as survival rate analysis, is employed to investigate patient survival time in relation to various factors and assess the correlation between survival time and outcomes, along with potential contributing factors. The KM methodology assesses the likelihood of a patient’s continued survival beyond a specific time frame, known as the survival probability. This probability is then multiplied by one to represent the corresponding survival rate for that particular period. We plotted the KM curve for ARDEGs(http://kmplot.com/ analysis) (27). *P*<0.05 was used as the cutoff for significance to find related genes.

***Confirming the expression of MAPK3, ERPP2 and BAX***

The differential expression of MAPK3, ERPP2, and BAX in normal and CESC tissues was verified through the analysis of the GSE63514, GSE52903, and GSE39001 datasets (*P*<0.05).

***Human protein atlas (HPA)***

HPA is a freely accessible, open database that provides researchers with the opportunity to explore human proteomes (28). In our study, we utilized immunohistochemistry to validate the protein expression of MAPK3, ERPP2, and BAX selected from both normal and CESC tissues. To obtain this information, we relied on the HPA database (http://www.proteinatlas.org/).

***GO, KEGG, and GSEA (co-expression genes of MAPK3 and BAX)***

We downloaded and collated the RNAseq data of the STAR process of the TCGA-CESC (cervical squamous cell carcinoma and adenocarcinoma) project from the TCGA database (https://portal.gdc.cancer.gov) (29) and extracted the TPM format data and clinical data to extract the corresponding molecules from the selected public data. The data were divided into high and low expression groups according to the expression of the corresponding molecules, and the DESeq2 (30) package was used to analyze the original Counts matrix of the selected public data according to the difference analysis. GO, KEGG, and GSEA enrichment analysis was performed by clusterProfiler [ 4.4.4 ] (26) and ggplot2 [ 3.3.6 ] R package.P.adj<0.05 and an FDR value (q.value) <0.05 were set as the cutoff criteria for significant enrichment.

***Immune infiltration analysis*** ***(*** ***TIMER and ssGSEA)***

Immune cell infiltration in different cancer types can be thoroughly assessed using the TIMER database. The association between MAPK3 expression and immune cell infiltration (dendritic cells, neutrophils, macrophages, CD4 + T cells, CD8 + T cells, and B cells) was examined using the database. Based on the ssGSEA algorithm provided in the R package-GSVA [ 1.46.0 ][31), the markers of 24 immune cells provided by the immunity article (32) were used to calculate the immune infiltration of the corresponding cloud data. The correlation between MAPK3 and immune infiltration matrix data was analyzed in TCGA-CESC, and the results were visualized by the ggplot2 package.

***Statistical analysis***

Data processing and analysis were performed using R software (Version 4.1.2). Mann-Whitney U test (also known as Wilcoxon rank sum test) was used to compare two groups of continuous variables with non-normal distribution and to evaluate the statistical significance of variables with normal distribution. The chi-square test or Fisher’s exact test was used to compare the statistical significance of the two groups of categorical variables. The significance level of *P*<0.05 was used to determine the statistical significance. All *P*-values were tested by two-sided test. The results were obtained using Spearman correlation analysis as the correlation coefficient between different compounds.

## Results

***Date procession***

**Figure 1:**
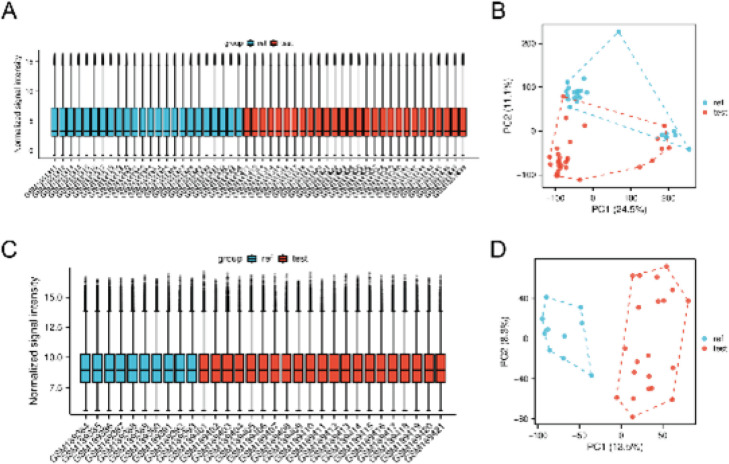
Sample distribution box plot and PCA plot show the correction of samples and the difference between samples

We standardized the GSE63514 and GSE7803 datasets, and the distribution box plot and PCA (Principal Component Analysis) plot (Figure 1A-D) displayed the standardized results.

***Cervical cancer-related DEGs***

**Figure 2:**
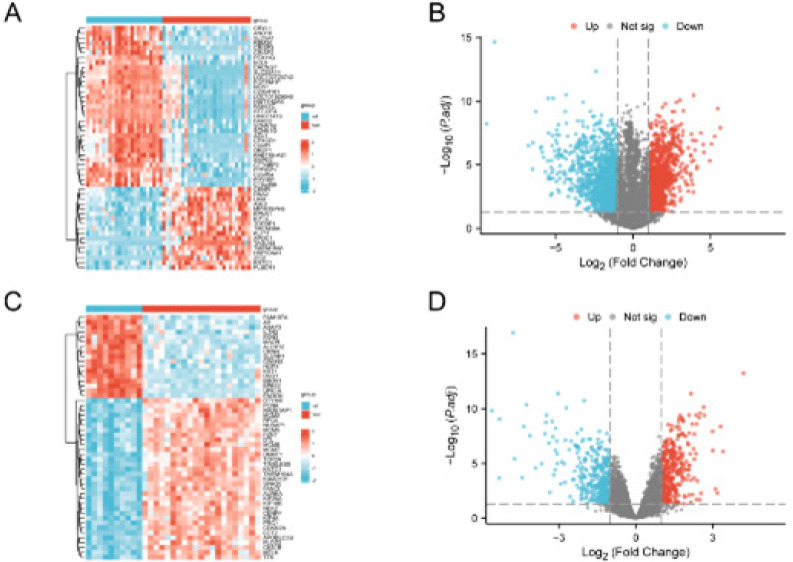
Heat map and volcano plot distribution of differential genes in the cervical cancer datasets

In order to identify differentially expressed genes (DEGs) between the Normal group and the CESC group, we used the Limma software package to evaluate the GSE63514 and GSE7803 datasets. The results are as follows: 2635 genes in the data set GSE63514 meet the |logFC|>1 and *P*<0.05 threshold. At this cut-off point, logFC > 1, there are 1243 up-regulated genes, logFC < -1, and 1392 down-regulated genes. We generated a heat map (Figure 2A) and a volcano map (Figure 2B) according to the results obtained from the differential analysis of this dataset. 729 genes in the data set GSE7803 meet the |logFC|>1 and *P*<0.05 threshold. Under this cutoff, logFC>1, there were 380 up-regulated genes, logFC<-1, and 349 down-regulated genes. We generated a heat map (Figure 2C) and a volcano map (Figure 2D) according to the results obtained from the differential analysis of this dataset.

**Figure 3:**
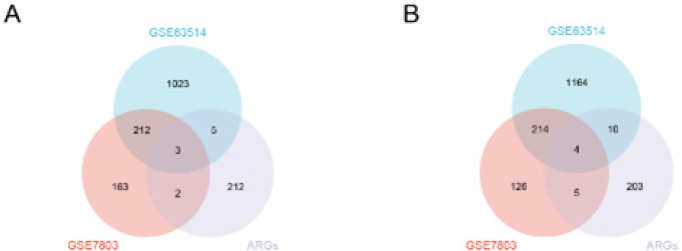
Venn diagram intersects the ARGs and DEGs of the GSE63514 and GSE7803 datasets to obtain ARDEGs

We intersected the up- and down-regulated DEGs of the GSE63514 and GSE7803 datasets with ARGs to produce ARDEGs. A total of 29 ARDEGs (Table 2) were obtained. The results were visualized using Venn diagrams (Figure 3A-B).

***GO and KEGG analysis of ARDEGs***

**Figure 4:**
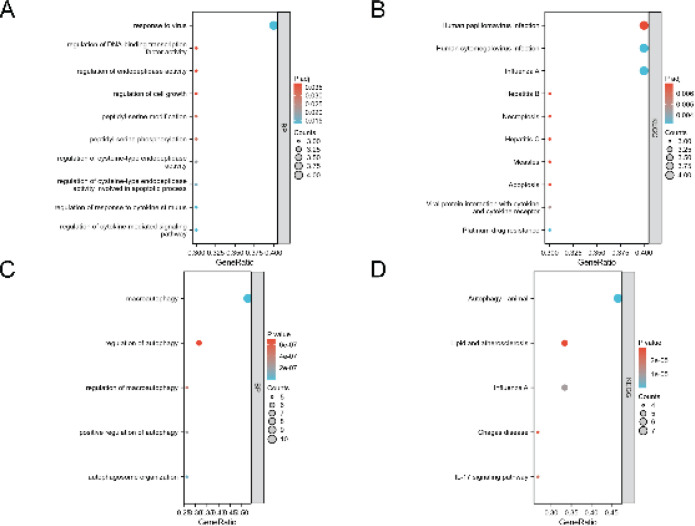
Results of functional enrichment analysis of ARDEGs

We performed GO and KEGG enrichment analysis of up-regulated and down-regulated ARDEGs to detect the biological processes of 29 ARDEGs (Tables 3, 4). We used a bubble diagram to show the results of GO and KEGG analysis. Through GO and KEGG enrichment analysis, we observed that the up-regulated ARDEGs showed significant enrichment in several biological processes. These included processes such as Response to virus (GO:009615), Regulation of cytokine-mediated signaling pathway (GO:0001959), Regulation of cysteine-type endopeptidase activity involved in the apoptotic process (GO:0043281), Regulation of cell growth (GO:0001558), Regulation of DNA-binding transcription factor activity (GO:0051090), and various other biological processes (Figure 4A). For KEGG, the up-regulated ARDEGs were mainly enriched in Human papillomavirus infection (hsa05165), Platinum drug resistance (hsa01524), Viral protein interaction with cytokine and cytokine receptor (hsa04061), Apoptosis (hsa04210), and Necroptosis (hsa04217)(Figure 4B). Down-regulated ARDEGs were mainly enriched in Regulation of autophagy (GO:0010506), Autophagosome organization (GO:1905037), Positive regulation of autophagy (GO:0010508), Regulation of macroautophagy (GO:0016241), Vacuole organization (GO:0007033) and other biological processes (Figure 4C). For KEGG, the down-regulated ARDEGs were mainly enriched in Autophagy-animal (hsa04140), IL-17 signaling pathway (hsa04657), TNF signaling pathway (hsa04668), Kaposi sarcoma-associated herpesvirus infection (hsa05167), and MAPK signaling pathway (hsa04010)(Figure 4D).

***Creating PPI networks and identifying hub genes***

**Figure 5:**
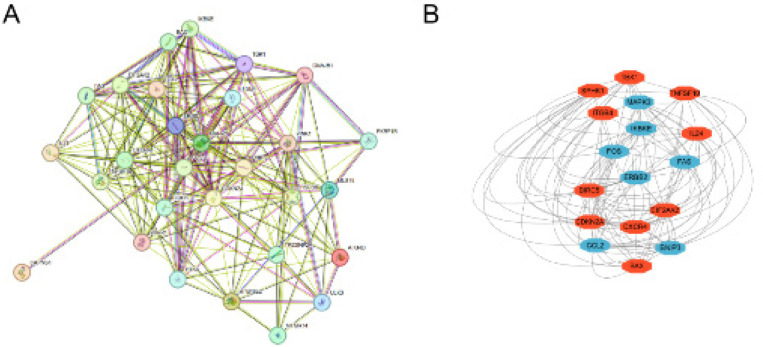
Validation of the correlation between genes extracted from the TCGA and GEO databases and identification of hub genes

A PPI network of ARDEGs was built using protein interaction analysis based on STRING networks. The PPI network (Figure 5A) comprises 162 edges and 29 nodes. With MCODE inserted into Cytoscape, 17 Hub genes were further identified (Figure 5B).

***Survival analysis of hub genes in cervical cancer***

**Figure 6:**
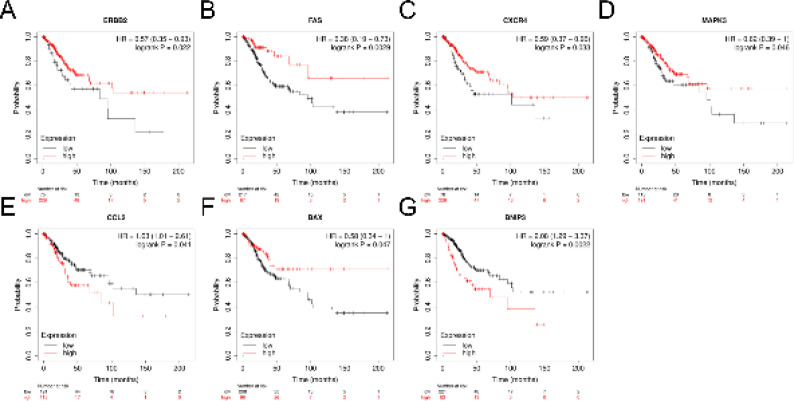
Prognostic analysis of 7 hub genes related to autophagy

Kaplan-Meier (KM) curves of 17 hub genes (ERBB2, FAS, CXCR4, TNFSF10, BIRC5, CDKN2 A, IKBKE, EIF2AK2, FOS, MAPK3, SPPK1, SPHK1, CCL2, BAX, IL24, BNIP3, TBK1, and ITGB4) were drawn through the database (http://kmplot.com/ analysis). Seven hub genes (ERBB2, FAS, CXCR4, MAPK3, CCL2, BAX, and BNIP3) were found to have statistically significant differences in OS (*P*<0.05, Figure 6A-G).

***Analysis of hub genes based on the TCGA-CESC database***

**Figure 7:**
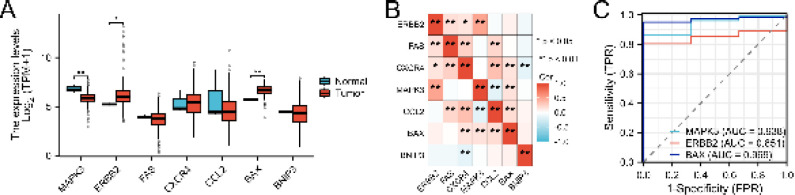
Validation of the expression difference, correlation, and diagnostic value of 7 hub genes based on TCGA-CESC databases

 Based on TCGA-CESC databases, the Wilcoxon rank sum test was used to analyze the difference of 7 hub genes between high and low groups by stats package and car package. The results showed that MAPK3, ERBB2, and BAX genes had significant expression differences in TCGA-CESC database (Figure 7A). The correlation analysis of 7 hub genes (ERBB2, FAS, CXCR4, MAPK3, CCL2, BAX, and BNIP3) in the data showed that MAPK3, ERBB2 and BAX were significantly correlated (Figure 7B). ROC analysis of MAPK3, ERBB2 and BAX genes was performed using the pROC package in TCGA-CESC databases. We found that MAPK3 and BAX have high diagnostic values, and ERBB2 has a certain diagnostic value (Figure 7C).

***Validation of MAPK3, ERBB2, and BAX expression based on various databases***

**Figure 8:**
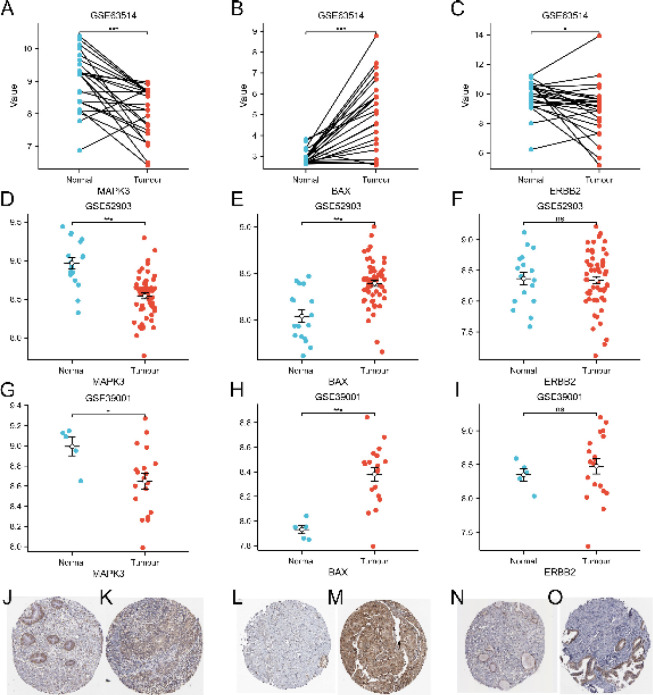
**.** Expression level of MAPK3, ERBB2, and BAX in cervical cancer

The expression differences of MAPK3, ERBB2, and BAX were further verified in GSE63514, GSE52903, and GSE39001 datasets. The results showed that MAPK3, ERBB2, and BAX had significant expression differences in the GSE63514 data set of paired samples (Figure 8A-C). The expression of MAPK3 and BAX in GSE52903 and GSE39001 datasets were significantly different, while the expression of ERBB2 was not significantly different (Figure 8D-I). HPA immunohistochemistry in the database further confirmed the differences in the expression of MAPK3, ERBB2, and BAX (Figure 8J-O).

***Analysis of MAPK3 and BAX co-expressed genes***

**Figure 9:**
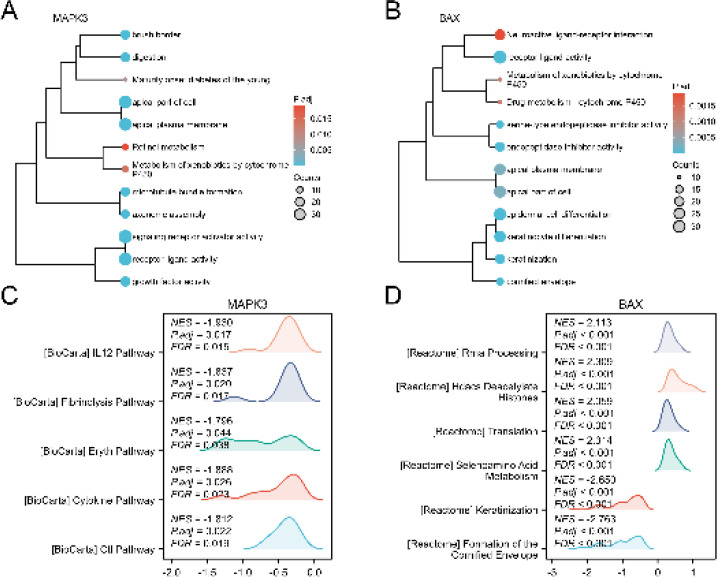
GO, KEGG, and GSEA enrichment analysis of MAPK3 and BAX co-expressed genes

To analyze the BP, MF, CC, and biological pathways of the MAPK3 and BAX Co-expressed genes, we first performed GO, KEGG, and GSEA enrichment analyses. Results showed that MAPK3 Co-expressed genes were mainly enriched in the signaling receptor activator activity (GO:0030546), growth factor activity (GO:0008083), Metabolism of xenobiotics by cytochrome P450(hsa00980), and other biological functions (Figure 9A). BAX Co-expressed genes were mainly enriched in the receptor-ligand activity (GO:0048018), Drug metabolism - cytochrome P450(hsa00982), Metabolism of xenobiotics by cytochrome P450hsa00980) and other biological functions (Figure 9B). Then we used GSEA to analyze the relationships between gene expression and biological processes, cellular components affected, and molecules involved. We found that MAPK3 and BAX Co-expressed genes in TCGA-CESC were significantly enriched in FCGR activation (NES=-2.524, p.adj=0.000, FDR=0.000; Figure 9B), immunoregulatory interactions between a lymphoid and a non-lymphoid cell, role of LAT2/NTAL/LAB calcium mobilization, role of phospholipids in phagocytosis, CD22 mediated BCR regulation, creation of C4 and C2 activators, Hdacs Deacetylate, Translation and Selenoamino Acid Metabolism (Figure 9C-D). These results imply that the development of CC may be influenced by immune-related pathways. Thus, additional investigation was conducted to explore the correlation between MAPK3 and BAX expression and immune cell infiltration in CC.

***Immune infiltration analysis (TIMER)***

**Figure 10:**
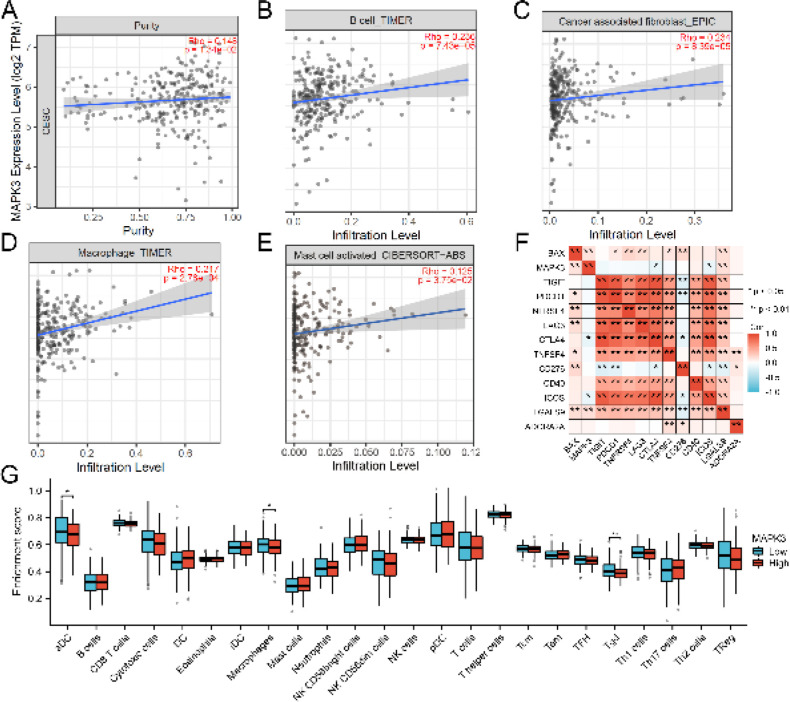
MAPK3 expression is related to immune cell infiltrations in cervical cancer

We further explored the association between immune infiltration levels and MAPK3 expression in CC by utilizing the TIMER database. Our results showed that the expression of MAPK3 was significantly positively correlated with the infiltration levels of macrophages (*P*=2.78 × 10-4), B cells (*P*=7.43 × 10-5), mast cell activation (*P*=7.43 × 10-5), and cancer-associated fibroblasts (*P*=8.39 × 10-5) (Figure10A-E). The correlation between BAX, MAPK3 and immune checkpoint genes (TIGIT, PDCD1, TNFRSF4, LAG3, CTLA4, TNFSF4, CD276, CD40, ICOS, LGALS9, and ADORA2A) was further analyzed. We found that BAX was positively correlated with TNFRSF4, LAG3, CD276, LGALS9, TIGIT, and TNFSF4. MAPK3 was positively correlated with LGALS9, but negatively correlated with CTLA4 and CD40 (Figure 10F). Then we further analyzed the differences in tumor-infiltrating immune cells between the MAPK3 high expression group and the low expression group, and found that there were significant differences in the expression of aDC, macrophages, and Tgd between the two groups (Figure10G). These findings demonstrated that MAPK3 is crucial to the immune infiltration of CC.

**Table 1:** Cervical squamous cell carcinoma (CESC) dataset information list used for analysis in this study

	TCGA-CESC	GSE63514	GSE7803		GSE52903		GSE39001
Platform		GPL570		GPL96		GPL14951	GPL6244
Species	Homo sapiens	Homo sapiens		Homo sapiens		Homo sapiens	Homo sapiens
Samples in Normal group	3	24		10		17	5
Samples in CESC group	309	28		21		55	19
Reference	[14]	[16]		[17]		[18]	[19]

(A) GSE63514 dataset sample distribution box plot and (B) PCA plot. (C) GSE7803 datasetsample distribution box plot and (D) PCA plot.

(A) Heat map of the GSE63514 dataset of DEGs. (B) Volcano plot of the GSE63514 dataset of DEGs. (C) Heat map of the GSE7803 dataset of DEGs. (D) Volcano plot of the GSE7803 dataset of DEGs.

**Table 2:** The up- and down-regulated DEGs between GSE146483 and GSE23558 microarray data

Category	ARDEGs
Up-regulated	CXCR4, BAX, SPHK1, EIF2AK2, TBK1, TNFSF10, ITGB4, CDKN2A, IL24, BIRC5
Down-regulated	PINK1, ATG16L2, ATG4D, IKBKE, MLST8, MAPK3, ERBB2, ULK3, TP53INP2, MTMR14, BNIP3, FOS, CCL2, FAS, CAPNS1, HSPB8, PTK6, FKBP1B ,DNAJB1

(A) ARGs and up-regulated DEGs of the GSE63514 and GSE7803 datasets. (B) ARGs and down-regulated DEGs of the GSE63514 and GSE7803 datasets.

**Table 3:** GO and KEGG enrichment analysis results of up-regulated ARDEGs

Ontology	ID	Description	GeneRatio	BgRatio	*P*-value	p.adjust
BP	GO:0009615	response to virus	4/10	392/18800	3.54e-05	0.0131
BP	GO:0001959	regulation of cytokine-mediated signaling pathway	3/10	166/18800	7.75e-05	0.0131
BP	GO:0060759	regulation of response to cytokine stimulus	3/10	178/18800	9.54e-05	0.0131
BP	GO:0043281	regulation of cysteine-type endopeptidase activity involved in apoptotic process	3/10	204/18800	0.0001	0.0163
BP	GO:2000116	regulation of cysteine-type endopeptidase activity	3/10	229/18800	0.0002	0.0197
BP	GO:0018105	peptidyl-serine phosphorylation	3/10	315/18800	0.0005	0.0292
BP	GO:0018209	peptidyl-serine modification	3/10	338/18800	0.0006	0.0305
BP	GO:0001558	regulation of cell growth	3/10	415/18800	0.0011	0.0374
BP	GO:0052548	regulation of endopeptidase activity	3/10	426/18800	0.0012	0.0374
BP	GO:0051090	regulation of DNA-binding transcription factor activity	3/10	452/18800	0.0015	0.0374

GO: Gene ontology analysis; KEGG: Kyoto encyclopedia of genes and genomes; ARDEGs: Autophagy-related differentially expressed genes

**Table 4:** GO and KEGG enrichment analysis results of down-regulated ARDEGs

Ontology	ID	Description	GeneRatio	BgRatio	*P*-value	p.adjust
KEGG	hsa05164	Influenza A	4/10	171/8164	3.54e-05	0.0032
KEGG	hsa05163	Human cytomegalovirus infection	4/10	225/8164	0.0001	0.0032
KEGG	hsa05165	Human papillomavirus infection	4/10	331/8164	0.0005	0.0069
KEGG	hsa01524	Platinum drug resistance	3/10	73/8164	7.87e-05	0.0032
KEGG	hsa04061	Viral protein interaction with cytokine and cytokine receptor	3/10	100/8164	0.0002	0.0046
KEGG	hsa04210	Apoptosis	3/10	136/8164	0.0005	0.0069
KEGG	hsa05162	Measles	3/10	139/8164	0.0005	0.0069
KEGG	hsa05160	Hepatitis C	3/10	157/8164	0.0008	0.0069
KEGG	hsa04217	Necroptosis	3/10	159/8164	0.0008	0.0069
KEGG	hsa05161	Hepatitis B	3/10	162/8164	0.0008	0.0069

GO: Gene ontology analysis; KEGG: Kyoto encyclopedia of genes and genomes; ARDEGs: Autophagy-related differentially expressed genes

(A) GO analysis of up-regulated ARDEGs and (B) KEGG analysis of up-regulated ARDEGs. (C) GO analysis of down-regulated ARDEGs and (D) KEGG analysis of down-regulated ARDEGs. Bubble diagram shows the results. *P*<0.05 and Q<0.05 were used as screening criteria.

(A) The PPI network represents the overlapping ARDEGs. (B) In Cytoscape, the MCODE algorithm was used to identify and confirm 17 hub genes.

(A-G) KM curve showing OS of ARDEGs. Black represents the Low-expression group, while red represents the High-expression group. OS, Overall survival.

(A) Expression level of 7 hub genes in the TCGA-CESC database. (B) Correlation analysis of 7 hub genes. (D) ROC analysis of MAPK3, ERBB2, and BAX genes.

(A-F) Expression level of MAPK3, ERBB2, and BAX based on the GSE63514, GSE52903, and GSE39001 datasets. Protein levels of MAPK3, ERBB2, and BAX in (J, L, N) cervical normal tissue (staining: medium; intensity: moderate, and quantity:75%–25%), and (K, M, O) CC tissue (staining: medium; intensity: moderate; and quantity: >75%).

(A-B) GOKEGG-clustering tree. (C-D) GSEA enrichment analysis of mountain map.

(A-E) We conducted a correlation analysis to assess the association between MAPK3 expression and immune cells. (F) Correlation between BAX, MAPK3, and immune checkpoint genes in the TCGA-CESC database. (G) Expression of immune cells infiltrating tumors among individuals with high and low MAPK3 expression in the TCGA-CESC database. (*P<0.05, ** *P*<0.01, and *** *P*<0.001).

## Discussion

CC is a highly prevalent malignant tumor in women and is the fourth leading cause of cancer-related death in this population. The prognosis of patients with metastatic or recurrent CC remains dismal, although new therapies including immunotherapy and vascular targeted therapy combined with radiotherapy and chemotherapy have improved survival rates. Therefore, it is crucial to study new molecular biomarkers and therapeutic targets to support the early identification, prevention, and treatment of CC. Autophagy is an important cellular process that enables cells to adapt to changing environmental conditions and respond to different stressors. Previous studies have shown that autophagy acts as a tumor suppressor in the early stages of tumorigenesis. However, with the development of the disease, its role has shifted to promoting the progression of cancer (33). Previous studies have shown that abnormal autophagy activity is closely related to the development of CC (34–37). However, the function of autophagy in CC has not been fully elucidated. Therefore, our study used bioinformatics to identify ARGs and evaluated their biological function in CC.

In this study, we identified 29 ARDEGs from TCGA and GEO databases, of which 10 were up-regulated and 19 were down-regulated. We then investigated the possible molecular processes of ARDEGs using GO and KEGG enrichment analysis. The ARDEGs were mainly enriched in platinum drug resistance, regulation of cell growth, regulation of DNA-binding transcription factor activity, human papillomavirus infection, positive regulation of autophagy, regulation of macroautophagy, IL-17 signaling pathway, TNF signaling pathway, and MAPK signaling pathway, etc. These signaling pathways are potentially related to the tumorigenesis and progression of CC (38–40). For example, IL-17A, as a mediator of inflammation-related cancer, promotes the progression of several malignancies, including breast cancer (41), colorectal cancer (42), lung cancer (43), and CC (44). The dysregulation and activation of the MAPK1/3 signaling pathway have been strongly correlated with the initiation and progression of diverse tumors (45–47), including but not limited to lung cancer (48), breast cancer (49), and liver cancer (50). In addition, autophagy is considered to be associated with tumor treatment resistance, consistent with our research (51). Therefore, GO and KEGG enrichment analysis showed that these ARDEGs were associated with CC progression.

Subsequently, we constructed a PPI network and identified 17 hub genes, of which 7 genes (ERBB2, FAS, CXCR4, MAPK3, CCL2, BAX, and BNIP3) were associated with OS of CC (*P*<0.05). The outcomes of the mRNA analysis and immunohistochemistry confirmed that the expression levels of BAX and MAPK3 were higher and lower, respectively, in those with CC tissues and normal tissues. MAPK3 is one of the crucial members of the MAPK family. A large number of studies have shown that a series of proteins produced by oncogenes promote the occurrence, development, and angiogenesis of tumors by continuously activating MAPK1/3 (52, 53). Moreover, our data show that decreased expression of MAPK3 is a poor prognostic biomarker for OS in CC and has high diagnostic value. Similarly, a number of earlier investigations have demonstrated that MAPK3 expression may be a possible prognostic indicator of reduced survival in patients with malignant tumors, including breast cancer (54), oral squamous cell carcinoma (55), and prostate cancer (56) are associated with MAPK1/3. We further analyzed 7 hub genes and found that there was a strong correlation between MAPK3 and BAX. BAX, a member of the pro-apoptotic Bcl-2 family, is dormant in the cytoplasm (57). A number of mitochondrial apoptotic proteins can be released into the cytoplasm by BAX, initiating the apoptosis cascade (58). Thus, we hypothesized that MAPK3 may regulate tumor apoptosis and autophagy by affecting BAX.

However, these results have not fully elucidated the potential mechanism of MAPK3 in CC, and the biological function and signaling pathway of MAPK3 need to be further explored. In this study, we performed GSEA and found that the significantly enriched pathways in MAPK3 expression included FCGR activation, immunoregulatory interactions between different types of cells, CD22-mediated BCR regulation, and the creation of C4 and C2 activators. These results suggest that MAPK3 plays a crucial role in the recruitment and regulation of immune cell infiltration in CC.

Autophagy and immunity are closely related, according to earlier research (59, 60). Furthermore, it has been demonstrated that invading immune cells can forecast the outcome of immune checkpoint inhibition (ICI) therapy and neoadjuvant chemotherapy (61). Therefore, screening infiltrating immune cells in CC can not only assist ICI treatment but also have potential predictive value for ICI treatment. As a result of the enrichment of MAPK3 expression in signaling pathways that regulate immune responses, we computed the correlation between MAPK3 expression and immune infiltration cell levels. This showed that MAPK3 expression was connected to the infiltration of activated mast cells, B cells, macrophages, and fibroblasts associated with cancer. Mast cells are one of the earliest immune cells to be recruited to solid tumors and are associated with angiogenesis during the early stages of cervical carcinogenesis (62). Regulatory B cells are related to the progression and metastasis of CC patients (63). These results suggest that MAPK3 expression may affect the progression and prognosis of CC by regulating the level of infiltrating immune cells.

Although the study contributes to our understanding of the relationship between MAPK3 expression and the prognostic value of CC patients, some limitations should be considered. In terms of sample size, TCGA and GEO data are insufficient. It is necessary to get more data. It is not enough to use only bioinformatics methods, and further *in vivo *and* in vitro *experiments are needed.

## Conclusion

This study reveals that MAPK3 is a marker for the diagnosis and prognosis of CC, and is closely related to immune infiltration. Our results imply that MAPK3 may be a potential prognostic biomarker for forecasting patient outcomes. However, more information is needed to fully understand how MAPK3 controls the occurrence and progression of CC.

## Authors' Contributions

SZ L designed the study; SZ L and K G collected and preliminary analyzed data. RY S interpreted the data; SZ L drafted the manuscript. DS Y made some modifications; DS Y was in charge of the entire study. The final draft was read and approved by all authors.

## Data Availability

The datasets included in this study can be downloaded from public repositories including the UCSC Xena database (http://genome.ucsc.edu), the TCGA Database (https://portal.gdc.cancer.gov/, and the GeneCards Database (https://www.genecards.org/ ).

## Funding Statement

1.Guangxi Natural Science Foundation Project (2022GXNSFAA035648)

2.Guangxi Zhuang Autonomous Region Health Commission self-funded research project (Z20210600)

3.Guangxi Medical and Health Appropriate Technology Development and Application Project (S2020096)

## Conflicts of Interest

The authors declare no conflicts of interest in this manuscript.
